# Controllable synthesis of nickel sulfides integrated with carbon fibers towards enhanced hydrogen evolution reaction kinetics

**DOI:** 10.1039/d5na00797f

**Published:** 2026-03-05

**Authors:** Yuting Li, Juan Wang, Qin Zhong

**Affiliations:** a College of Safety Engineering and Emergency Management, Nantong Institute of Technology Nantong Jiangsu 226002 China; b School of Chemistry and Chemical Engineering, Nanjing University of Science and Technology Nanjing Jiangsu 210094 China wangjuan304@njust.edu.cn zq304@njust.edu.cn

## Abstract

Nickel sulfides are considered as one of the promising electrocatalysts for the hydrogen evolution reaction (HER). Herein, the monolith NiS_*x*_@CNFs was constructed as a HER working electrode *via in situ* electrospinning, and the crystal phase of Ni_9_S_8_ and NiS could be controlled by adjusting the annealing temperature and S source. Specifically, NiS-800@CNFs exhibits excellent activity with the required overpotential of 119 mV at 10 mA cm^−2^. This is attributed to the sulfur-deficient Ni_9_S_8_ crystal that provides sufficient hydrogen adsorption sites, with the coexistence of the NiO phase that is beneficial for water dissociation, which synergically promotes the alkaline HER process. Based on the electrochemical impedance spectroscopy (EIS) characterization, the HER mechanisms of the NiS_*x*_@CNFs series were systematically explored. It is revealed that the sulfur-rich NiS crystal surface is not conducive to the desorption of adsorbed hydrogen to produce hydrogen. This work provides a valuable reference for the regulation of phase structure and the HER mechanism of nickel sulfides.

## Abstract 2

Nickel sulfides were controllably synthesized *via* electrospinning, and their structure activity relationships and kinetic mechanisms of the HER were systematically discussed.

## Graphical abstract

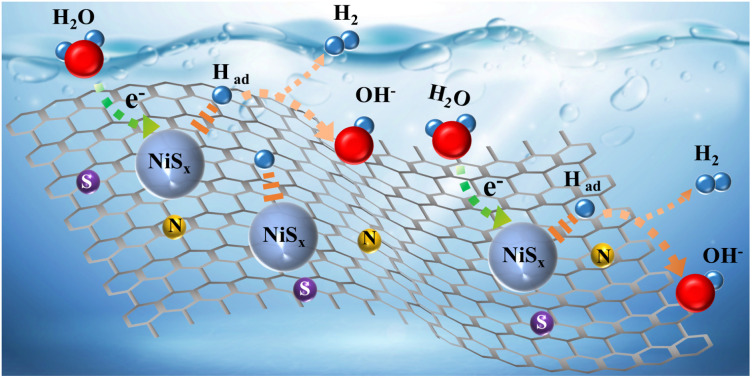

## 1 Introduction

With growing concerns about environmental pollution and energy consumption, hydrogen generation from electrochemical water splitting has become an ideal replacement for traditional fossil fuels on account of its high energy density and abundant raw resources.^1,2^ Alkaline water electrolysis (AWE) is one of the widely used green hydrogen production technologies.^3–5^ As the key electrode reaction of AWE, the electrocatalyst activity of the hydrogen evolution reaction (HER) plays an important role in this process. Elementary reaction of HER proceeds through the Volmer, Heyrovsky and Tafel reaction mechanism.^6–8^ In alkaline electrolytes, the H atom is generated from the decomposition of H_2_O into H^+^, which must overcome the water dissociation process; this step requires a higher reaction energy barrier compared with the HER in acidic media.^9^ Thus, the Volmer step of the HER to form the H atom adsorbed on the catalyst surface in alkaline electrolytes is crucial. An ideal HER catalyst under alkaline medium should meet the following two criteria: (i) the catalyst should reveal the function of activating and breaking the hydrogen bonding of water molecules to form H atoms, and even the H atoms should be easily adsorbed on the surface of catalyst (H_ads_) and (ii) H atoms cannot be strongly adsorbed on the catalyst surface, resulting in an unfavorable H_2_ desorption process.^10,11^ Therefore, optimizing the adsorption and desorption capacity of hydrogen atoms on the electrocatalyst surface is of great significance for efficient hydrogen evolution.

Transition metal sulfides (TMS) have been a popular electrocatalyst in the field of electrocatalysis due to their high surface area, unique electronic structure and abundant phase structure.^12,13^ In the process of water decomposition, the S–H_ads_ bond is easily generated on the surface of the metal sulfide catalyst, which is conducive to H adsorption. However, the S–H_ads_ bond on the surface is usually strong. The excessive interaction between S and adsorbed H makes it difficult for the desorption of H_ads_ to produce H_2_.^14,15^ Moreover, with the continuous progress of the HER, the adsorption site of the metal sulfide presents a saturated state, which makes it difficult to adsorb water molecules.^16,17^ All the above factors have an obvious inhibitory effect on the HER catalytic activity of metal sulfide. Fine-tuning the electronic structure of the catalyst could improve the adsorption/desorption binding energy of the reaction intermediates. Therefore, the adsorption and activation of water can be regulated by changing the electron density distribution of metal sulfides, and the adsorption and desorption of H can be optimized to promote the kinetic rate of the HER.^18,19^

Nickel sulfides (NiS_*x*_), one of the transition-metal sulfides, have long been considered as promising candidates for HER catalysts because of their low cost, earth abundance, easy preparation, and high catalytic activity.^20–22^ Given the previous reports, the atomic ratio of NiS_*x*_ can be varied across a wide range (*x* can be decreased from 2.0 to 0.67), such as NiS_2_, Ni_3_S_4_, NiS, Ni_9_S_8_, Ni_7_S_6_ and Ni_3_S_2_.^23–29^ Notably, the crystal structures and electronic properties of NiS_*x*_ can be significantly impacted by the modulation of phase and chemical composition, which is a promising opportunity for optimizing their electrocatalytic performance. The adsorption force of H also varies with the content of exposed S sites in metal sulfides.^30^ Although a large number of nickel sulfides have been reported as HER catalysts at present, the changes of different phases or heterogeneous structures of nickel sulfides and their corresponding structure–activity relationships and kinetic mechanisms are still unclear.

The studied nickel sulfides have been typically supported on conductive carriers (such as nickel foam, Ti plate, and carbon cloth) to serve as applied electrodes for electrochemical systems. However, such supporting electrodes could suffer from surface agglomeration, exfoliation and bubble accumulation during the HER process. Given our previous efforts, monolithic construction formed *via* the electrospinning method prevented the detachment of active sites and enhanced the conductivity of the catalysts.^31^ Herein, the monolith NiS_*x*_@CNFs series with different sulfur phases was prepared by *in situ* electrospinning and directly considered as working electrodes. By controlling the temperature and sulfur content, the atomic ratio of NiS_*x*_ was achieved at 0.88 to 1.0, including phases of Ni_9_S_8_ and NiS. Such monolith structures, integrating nickel sulfides and the conductive N- and S-doped carbon fibers, can result in efficient mass and electronic transports. With the increase in sulfur content, the catalyst phase changes from sulfur-deficient Ni_9_S_8_ crystal into sulfur-rich NiS crystal. NiS-800@CNFs consists of sulfur-deficient Ni_9_S_8_ crystal and exhibits excellent HER activity and stability at the high current density of 200 mA cm^−2^ for 50 h. Different crystal phases and electron structures influence the HER mechanism and rate determination step (RDS). Based on EIS analysis, the NiS crystal is beneficial for water decomposition to form absorbed H atoms, while the Ni_3_S_2_ and Ni_9_S_8_ crystals are conducive to H_2_ generation.

## 2 Experimental methods

### 2.1 Materials

Polyacrylonitrile (PAN), thiourea (CH_4_N_2_S), *N*,*N*-dimethylformamide (DMF) and Ni(NO_3_)_2_·6H_2_O served as original materials to prepare the nickel sulfide carbon nanofibers. All chemicals were directly used without further purification.

### 2.2 Synthesis of the precursor

1.1 g PAN, 1.0 mmol Ni(NO_3_)_2_·6H_2_O and 1.0 mmol thiourea were thoroughly dissolved in 10 mL of DMF. The mixture solution was subjected to electrospinning with a feeding rate of 0.02 mL mim^−1^ at an electrostatic voltage of 15 kV. The light blue fibrous precursor was collected.

### 2.3 Synthesis of NiS_*x*_@CNFs series

The above precursor was first heated at 200 °C for 5 h with a heating rate of 2 °C min^−1^ under a N_2_ atmosphere to gain pre-treated nanofiber flakes. The pre-treated nanofiber was subjected to two distinct synthesis methods to control the formation of different nickel sulfide phases.

#### 2.3.1 Synthesis of NiS-*T*@CNFs

The pre-treated flake was secondarily annealed at 500 °C, 550 °C, 600 °C, 700 °C, 800 °C for 2 h under a N_2_ atmosphere and heating rate of 2 °C, respectively. When cooled to room temperature, the obtained black samples were denoted as NiS-500@CNFs, NiS-550@CNFs, NiS-600@CNFs, NiS-700@CNFs, and NiS-800@CNFs, respectively. Here, NiS-T serves as a sample identifier.

#### 2.3.2 Synthesis of NiS-*y*@CNFs

The pre-treated flake and thiourea were placed onto a porcelain boat. The pre-treated flake was further subjected to sulfidation in N_2_ atmosphere at 500 °C for 1 h with a heating rate of 2 °C min^−1^. The thiourea was situated in the upstream of the gas flow. The dosage of thiourea was 0, 1 and 2 mmol, and the synthesized black samples were denoted as NiS-0@CNFs, NiS-1@CNFs and NiS-2@CNFs, respectively.

### 2.4 Characterization

The micro-morphology, phase and component structure of the NiS_*x*_@CNFs series were investigated by scanning electron microscopy, X-ray diffraction, Raman spectroscopy, nitrogen adsorption–desorption, X-ray photoelectron spectroscopy, and high-resolution transmission electron microscopy. The details of the structural characterizations are provided in the SI.

### 2.5 Electrochemical measurements

The electrochemical properties of the monolithic NiS_*x*_@CNFs series were studied in a three-electrode system in 1 M KOH electrolyte. The monolithic NiS_*x*_@CNFs directly served as working electrodes. The HER kinetics of the NiS_*x*_@CNFs series were analyzed *via* electrochemical impedance spectroscopy (EIS). EIS was measured at an open-circuit voltage, with frequencies ranging from 10^5^ Hz to 1 Hz at an AC amplitude of 5 mV. The details of electrochemical measurements are illustrated in the Supplementary Information. EIS was measured using an open-circuit voltage with frequencies ranging from 10^5^ Hz to 1 Hz at an AC amplitude of 5 mV.

## 3 Result and discussion

**Scheme 1:**
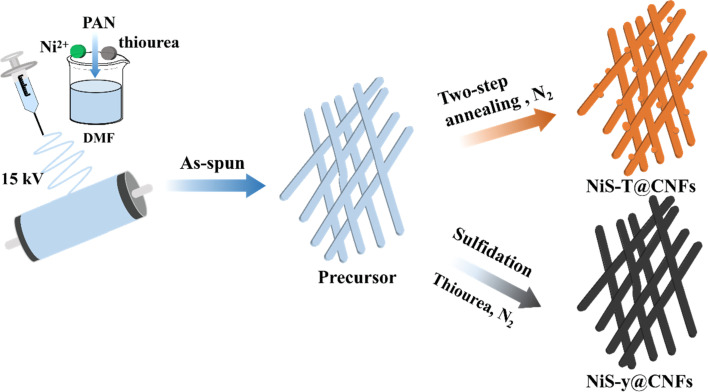
Schematic of the synthesis of the NiS_*x*_@CNFs series.

Scheme 1 presents the synthesis process of monolithic NiS_*x*_@CNFs series. The precursor achieved *via in situ* electrospinning method was pre-treated first under a low temperature, and subjected to secondary carbonization and further sulfidation treatment, respectively. By adjusting the annealing temperature and sulfur content (yielding NiS-*T*@CNFs and NiS-*y*@CNFs), it is aimed to control the formation of different phases of nickel sulfides, which allows for a systematic comparison of how phase composition and synthesis strategy influence the microstructure and performance of the material. Through a multi-annealing step, the intertwined fibers endow internal mechanical strength and build the self-supporting construction of NiS_*x*_@CNFs catalysts that served as monolithic electrodes.

**Fig. 1:**
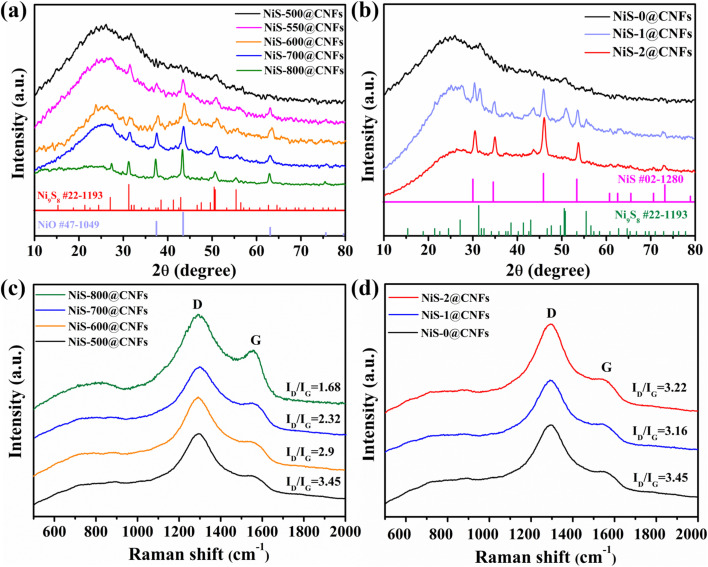
XRD spectra of NiS-*T*@CNFs (a) and NiS-*y*@CNFs (b). Raman spectra of NiS-*T*@CNFs (c) and NiS-*y*@CNFs (d).

The phase structure of nickel sulfide under different process conditions was observed by XRD. Fig. 1a and b present the XRD patterns of the catalysts under diverse secondary annealing temperatures and different sulfur source contents. As shown in Fig. 1a, the wide peak at 26° corresponds to the (002) plane of the carbon peak. With the increasing temperature, peaks at 27.2°, 31.5°, 50.8° and 55.6° gradually appear and mainly correspond to the (202), (222), (025) and (530) crystal planes, respectively, of the sulfur-poor phase Ni_9_S_8_ (JCPDS: 22-1193). In addition, peaks at 37.5°, 43.4°, and 63° are mainly attributable to the (111), (200), and (220) crystal planes, respectively, of the NiO phase (JCPDS: 47-1049). These results reveal that the annealing temperature has no effect on the phase structure of nickel sulfide. The higher the annealing temperature, the sharper the peaks of Ni_9_S_8_ and NiO become, indicating enhanced crystallinity. As shown in Fig. 1b, the Ni_9_S_8_ phase peaks of NiS-1@CNFs become stronger than those of NiS-0@CNFs. Meanwhile, the evident peaks at 30.2°, 35°, 46°, 53.7°, and 73.3° in NiS-1@CNFs correspond to the (100), (101), (102), (110), and (202) crystal faces, respectively, of the NiS phase (JCPDS: 02-1280). However, the Ni_9_S_8_ peaks in NiS-2@CNF disappear and the NiS phase peaks become obvious. These results show that the sulfur-deficient phase Ni_9_S_8_ gradually changes to the sulfur-rich phase NiS, with the increase of sulfur partial pressure and the set temperature of 500 °C, which proves an effective control strategy for achieving species transformation of nickel sulfide. Fig. 1c and d depict the Raman spectra of the NiS_*x*_@CNFs series. The peaks at 1294 and 1554 cm^−1^ reflect the D and G bands of disordered carbon and graphite sp^2^ carbon.^32^ As observed in Fig. 1c, the *I*_D_/*I*_G_ values of NiS-*T*@CNFs are greater than 1.00, indicating the presence of defective carbon. With the increase in annealing temperature, the value of *I*_D_/*I*_G_ becomes smaller, indicating that the NiS-800@CNFs carbon structure is gradually graphitized at high temperature. During the process of high temperature carbonization, Ni salt decomposes and combines with S to form Ni_9_S_8_, which is beneficial to further promoting the formation of graphitic carbon. In Fig. 1d, with the increase of sulfur content, the trend of *I*_D_/*I*_G_ value is not obvious, proving that the transition of the nickel sulfide phase structure has no influence on the carbon structure.

**Fig. 2:**
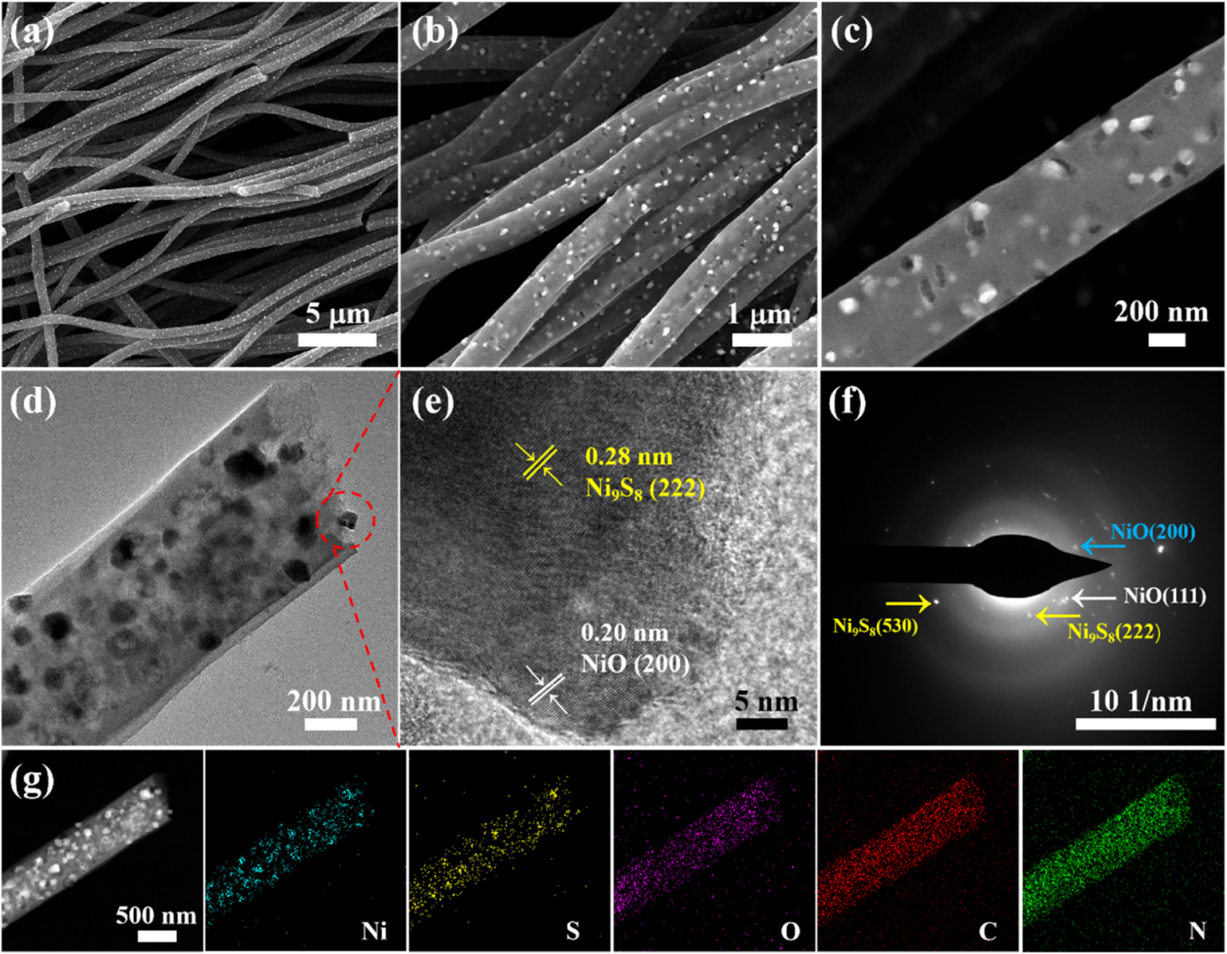
(a–c) SEM images of NiS-800@CNFs at different magnifications. TEM (d) and HRTEM (e) images and SAED pattern (f) of NiS-800@CNFs. (g) Elemental mapping images of NiS-800@CNFs.

The fiber micro-structures of NiS_*x*_@CNFs samples were investigated *via* SEM and HR-TEM characterizations. Firstly, Fig. 2a shows that the NiS-800@CNFs nanofibers cris-cross and randomly interweave to form a network structure. In Fig. 2b and c, it can be clearly observed that a single bundle of nanofiber exhibits a porous structure and is covered with numerous nanoparticles on the surface. This pore system can facilitate efficient electrolyte infiltration and expose a vast number of accessible active sites to the reactants. Fig. 2d further reveals the presence of well-dispersed nickel sulfide nanoparticles embedded within the porous carbon fiber matrix. As shown in Fig. 2e and f, the HRTEM image and corresponding SAED pattern prove a polycrystalline structure and the co-existence of Ni_9_S_8_ and NiS species, consistent with the XRD result. Elemental mapping images demonstrate the successful formation and uniform distribution of nickel sulfide (Fig. 2g).

**Fig. 3:**
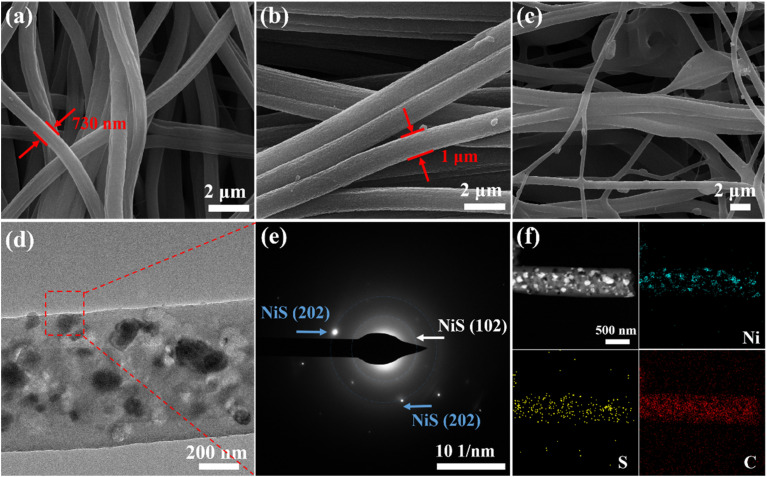
SEM images of (a) NiS-0@CNFs, (b) NiS-1@CNFs and (c) NiS-2@CNFs. TEM image (d) and SAED pattern (e) of NiS-2@CNFs. Elemental mapping images (f) of NiS-2@CNFs.

Differently, as shown in Fig. 3a–c, NiS-*y*@CNFs under different thiourea contents do not show obvious voids and nanoparticles on the surficial carbon fiber. Especially, Fig. 3c shows that the fibre sizes are uneven and the partial fibers of NiS-2@CNFs blend together to form thick nanobundles. In Fig. 3d and e, it can be demonstrated that the NiS nanoparticles are all embedded within the carbon fiber of NiS-2@CNFs, and the fiber interior also has a porous structure. Elemental mapping images further demonstrate the successful sulfidation (Fig. 3f). Fig. S1 shows the EDX spectra of NiS-*y*@CNFs samples and demonstrates the existence of mainly C, N, O, Ni and S elements. With the increasing content of thiourea, the weight ratio of Ni to S decreases from 26.2 to 2.8, which is partly related to the conversion of sulfur-deficient phase Ni_9_S_8_ into sulfur-rich phase NiS. Fig. S2a shows the isotherm curve of nitrogen absorption/desorption of NiS-*y*@CNFs. The hysteresis loops at the relative pressure of *P*/*P*_0_ at 0.5–1.0 indicate the existence of a mesoporous structure, which may be caused by the volume expansion of metal sulfide at high temperature. The pore size distribution curve shows that most pore sizes are distributed between 2 and 5 nm (Fig. S2b), demonstrating the co-existence of microporous and mesoporous structures. Compared with NiS-0@CNFs, the micropore structure of NiS-1@CNFs is concentrated at 2 nm. Therefore, NiS-1@CNFs exhibits a large specific surface area, which is conducive to the exposure of more active sites and promoting charge and reactant transport.

**Fig. 4:**
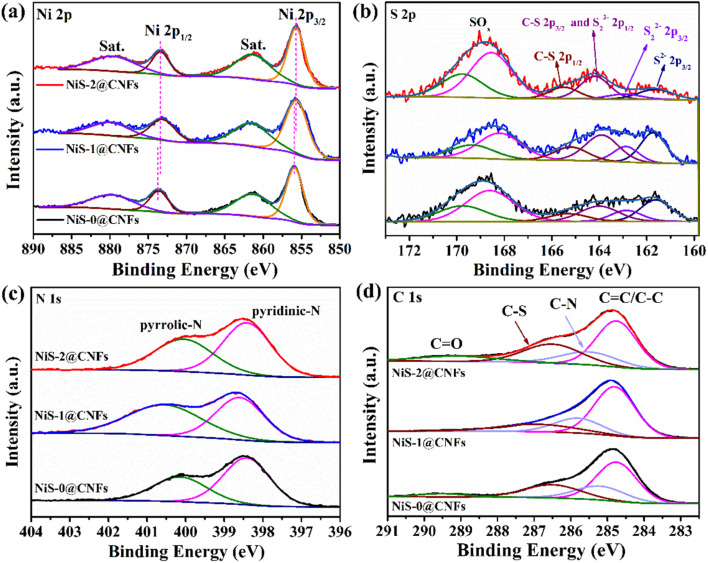
XPS spectra of Ni 2p (a), S 2p (b), N 1s (c) and C 1s (d) for NiS-*y*@CNFs.

In order to further study the valence states of elements with nickel sulfur phases, the XPS spectra of NiS-*x*@CNFs is shown in Fig. 4. As shown in the Ni 2p spectrum (Fig. 4a), the characteristic peaks at 855.9 and 873.6 eV belong to Ni^2+^ 2p_3/2_ and Ni 2p_1/2_, respectively. The peaks at 861.2 and 879.7 eV correspond to the satellite peaks of Ni. Moreover, with the increase in thiourea content, the Ni 2p of NiS-2@CNFs shifts toward a lower binding energy, indicating that the phase transition affects the surface electron cloud density. The increase in electron cloud density on the surface of Ni in NiS-2@CNFs can enhance the adsorption of H and promote the alkaline water splitting process.^33^ In the S 2p spectrum (Fig. 4b), the peaks at 161.7 and 162.9 eV correspond to S 2p_3/2_ of S^2−^ and S 2p_1/2_ of S^2−^ and S_2_^2−^. The peaks at 164.2 and 165.5 eV correspond to S_2_^2−^ 2p_1/2_, S 2p_3/2_ of C–S, and S 2p_1/2_ of C–S, respectively.^34,35^ Compared with NiS-0@CNFs and NiS-2@CNFs, the large peak areas of S_2_^2−^ 2p and S^2−^ 2p at 162.8 eV for NiS-1@CNFs are mainly attributed to the presence of multi-component Ni_9_S_8_ and NiS heterostructures. In terms of NiS-2@CNFs, the peak of S^2−^ 2p weakens while the peak of S_2_^2−^ 2p_1/2_ enhances. The excess S exists in the form of bridging S_2_^2−^, indicating the presence of unsaturated S atoms in the catalyst.^34,36,37^ Typical characteristic peaks of pyridine nitrogen (398.5 eV) and pyrrole nitrogen (400 eV) are shown in the N 1s spectrum of NiS-*x*@CNFs (Fig. 4c). As shown in Fig. 4d for the C 1s spectrum, the peaks at 284.7, 285.5 and 286.5 eV correspond to the fitting peaks of C=C/C–C, C–N and C–S,^38^ respectively, indicating the S and N atoms were incorporated in the carbon base.

**Fig. 5:**
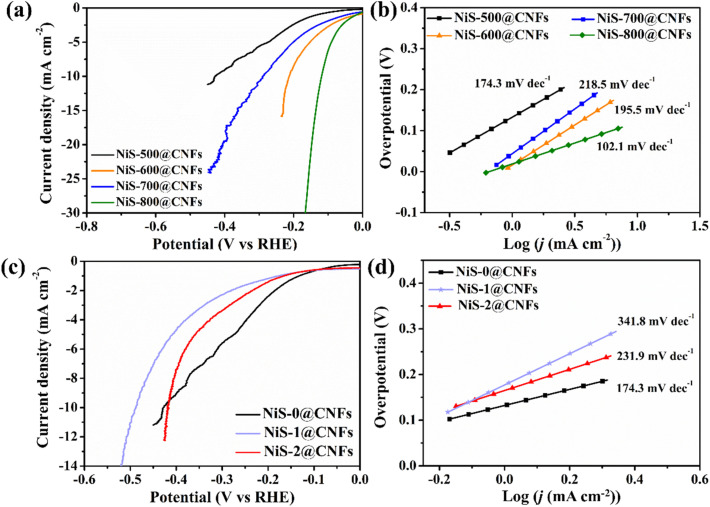
LSV curves (*iR*-corrected) of NiS-*T*@CNFs (a) and NiS-*y*@CNFs (c) for HER in 1 M KOH; corresponding Tafel slopes of NiS-*T*@CNFs (b) and NiS-*y*@CNFs (d)..

In order to investigate the influence of different phase structures of nickel sulfides on the electrochemical performance and HER mechanism, LSV curves and Tafel slopes of monolithic NiS-*T*@CNFs and NiS-*y*@CNFs electrodes are shown in Fig. 5. It can be found that NiS-800@CNFs exhibits the best HER performance with the overpotential of 119 mV at 10 mA cm^2^ and Tafel slope of 102.1 mV dec^−1^ (Fig. 5a and b), which is better than the reported self-supporting metal sulfides (Table S1). Based on the XRD peaks and SEM results (as shown in Fig. 1a and 2b), with the temperature increasing, the HER performance is improved in general due to the highly developed crystallinity of Ni_9_S_8_ and the well-preserved, interconnected porous structure that provides more exposed active sites of adsorption, thus promoting the HER process. As shown in Fig. 5c and d, the sulfur-deficient phase Ni_9_S_8_ (NiS-0@CNFs) showed better HER activity than NiS-2@CNFs and NiS-1@CNFs due to severe aggregation of nanofibers drastically reducing the number of accessible active sites according to the SEM image in Fig. 3c. Despite the Ni_9_S_8_ and NiS contents, the heterogeneous component of NiS-1@CNFs are not beneficial to the HER activity. According to the Tafel slope values, all NiS_*x*_@CNFs series samples follow the Volmer–Heyrovsky mechanism in alkaline electrolytes.

**Fig. 6:**
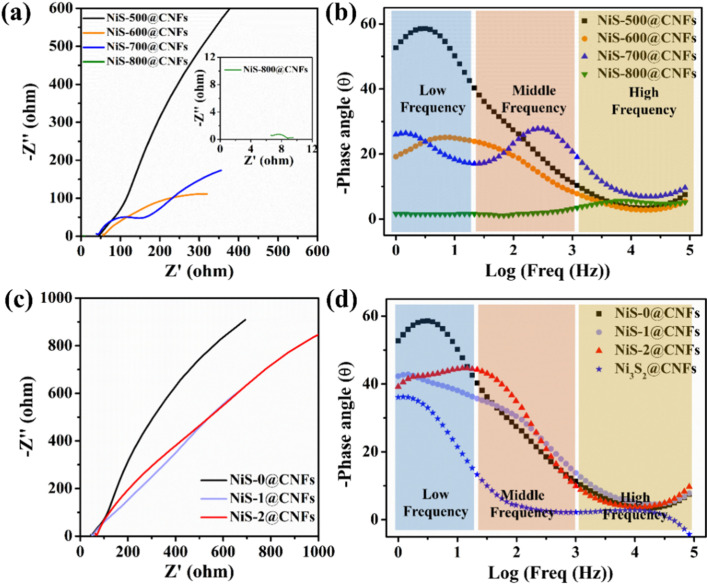
Nyquist plots of NiS-*T*@CNFs (a) and NiS-*y*@CNFs (c) under open-circuit voltage with frequency ranging from 10^5^ Hz to 1 Hz; corresponding Bode images of NiS-*T*@CNFs (b) and NiS-*y*@CNFs (d).

According to the Nyquist and corresponding Bode images in Fig. 6, the HER mechanism and the rate-determining step (RDS) were further analyzed and revealed according to the phase angles of responses in different frequency regions.^39^ The low-frequency process is usually associated with slower kinetic steps, such as those involving mass diffusion, interface adsorption processes and formation or transformation of adsorbates.^40^ Therefore, during the HER process, the low-frequency peak that predominates at low overpotentials can be classified as the Volmer step. In alkaline HER, the subsequent step is the Heyrovsky step, which is the electrochemical desorption to form H_2_ on the surface of the electrode. The middle-frequency region is related to the interface reaction charge transfer and double-layer capacitance between the electrode and electrolyte, which significantly influences the kinetic process of the formation and desorption process of H_2_ (Heyrovsky step). The high-frequency region often reflects rapid processes such as solution resistance and electrode contact resistance. As shown in Fig. 6b, the phase angle of NiS-500@CNFs responses only in the low frequency region, indicating that the accumulation process of reactive intermediates on the electrode surface is low and the formation of adsorbed hydrogen (Volmer step) is considered as the RDS. With the rise in temperature, the phase angle decreases in the low-frequency region primarily due to the facilitation of water splitting by NiO generation.^41^ The formation of Ni_9_S_8_ promotes hydrogen adsorption and collaboratively catalyzes the HER process. Consequently, NiS-700@CNFs exhibits a significant phase angle in the mid-frequency region, indicating that the interfacial charge transfer between the electrochemical surface layer of the catalyst and the electrolyte is relatively slow. It is attributed to the constantly emerging NiO and Ni_9_S_8_ at high temperatures leading to excessive accumulation of surface adsorbed reactants (H_2_O* and H*), thereby reducing the reaction rate of the Heyrovsky step. The fluctuation observed in the LSV curve for NiS-700@ CNFs at high currents indicates difficulty in the surface H_2_ desorption process. According to the Nyquist plot of NiS-800@CNFs (Fig. 6a), fitting the equivalent circuit achieved the *R*_s_ and *R*_ct_ values, as shown in Fig. S3. It can be found that the fitted Nyquist plot shows two semi-circular shapes. According to the equivalent circuit diagram, the *R*_ct_ value is 2.89 in the high-frequency semi-circular area, which corresponds to the prominent phase angle peak in the mid-high-frequency region of the Bode plot (Fig. S3b), indicating that the charge-transfer process is the dominant relaxation process. Thus, the HER kinetics on the catalyst surface is mainly influenced by charge transfer due to the poor electron transfer ability of NiO, despite that it can promote water dissociation.

As shown in Fig. 6c and d, with the transition to the NiS phase, the broad phase angle peaks mainly appear in low and middle frequency regions. As the overpotential is increased (in Fig. S4), the phase angle is reduced at the low frequency region, while phase angle peaks increase at the middle frequency region. High voltage accelerates the Volmer step, while the electrochemical desorption step (Heyrovsky step) is relatively slow. Particularly, the phase angle peak of NiS-2@CNF with sulfur-rich NiS is very prominent in the middle-frequency region under high voltage. The adsorption of H atom becomes strong, attributed to the formation of S_2_^2−^ by excess S on the surface of NiS-2@CNFs. It makes the formation and desorption process of H_2_ difficult, thus lowering the reaction rate of the Heyrovsky step. This also explains the decreased catalytic activity of NiS-1@CNFs and NiS-2@CNFs.

In addition, compared with the Ni_3_S_2_@CNFs of our previous study, the Bode curve of NiS-0@CNFs has a response peak in the low frequency region (Fig. 6d), which indicates that the dissociation of adsorbed water into adsorbed hydrogen is the RDS process. Therefore, the Volmer step of the sulfur-deficient phase (Ni_3_S_2_, Ni_9_S_8_) is difficult in the alkaline HER process, while the Heyrovsky process of the sulfur-rich phase NiS is slow.

**Fig. 7:**
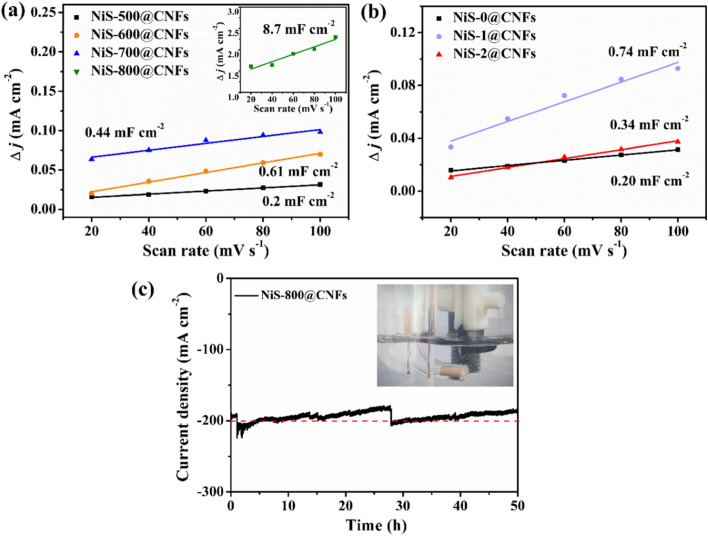
Double layer capacitance curves of NiS-*T*@CNFs (a) and NiS-*y*@CNFs (b). (c) Durability curve of NiS-800@CNFs at a high current density of 200 mA cm^−2^ (inset: hydrogen generation with the monolith NiS-800@CNF electrode).

The *C*_dl_ value of ECSA was calculated to further evaluate the intrinsic activity of the catalyst, as shown in Fig. 7. According to the fitting curve of NiS-*T*@CNFs in Fig. 7a, NiS-800@CNFs exhibits the highest *C*_dl_ value, proving a relatively high electrochemically active area. It is attributed to the synergy among the porous structures, large exposed Ni_9_S_8_ crystal and enhanced graphitization, ensuring maximum exposure and utilization of active sites. In Fig. 7b, NiS-1@CNFs shows the largest *C*_dl_ value, mainly due to the coexistence of multiple components Ni_9_S_8_ and NiS, which provide multiple active sites (Ni and S sites). However, although excessive S_2_^2−^ sites in NiS-2@CNFs can promote the deprotonation of water molecules,^42^ it is not conducive to the desorption of the intermediate state H* to form H_2_. Therefore, under a high current density, the adsorption layer of intermediate H* formed on the catalytic surface is enhanced, which is not conducive to further binding H_2_O to form H_2_. Furthermore, the HER stability of NiS-800@CNFs selected as the cathode material was evaluated, as shown in Fig. 7c. Numerous H_2_ bubbles are released on the surface of the catalyst and there is no gathered gas film, as shown in inset Fig. 7c. Overall, NiS-800@CNFs can maintain good durability. Although its performance decreases over the first 27 hours, the current density can still remain at around 200 mA cm^−2^ after replenishing the electrolyte.

After durability tests, the SEM, XRD and XPS characterizations of NiS-800@CNFs were conducted as shown in Fig. S5 and S6. The XRD pattern proves that NiS-800@CNFs still exhibits Ni_9_S_8_ and NiO phases after durability tests under a high current density, as shown in Fig. S5a. In addition, new diffraction peaks appear at 11.9°, 33.8° and 38.9°, corresponding to the (001), (110) and (200) crystal planes of Ni(OH)_2_, respectively, (JCPDS: 22-0444),^43,44^ which indicates a significant structural evolution of the catalyst. In Fig. S5b and c, SEM images show that the NiS-800@CNFs catalyst still retains a continuously interwoven network structure without obvious breakage. According to the XPS data of S and O elements (Fig. S6b and c), the characteristic peaks of S^2−^ 2p and Ni–O still exist, suggesting that the Ni_9_S_8_ and NiO components are retained after long-term stability tests, which benefited the carbon fiber framework, preventing the loss of active species. In Fig. S6a, the characteristic peaks at 855.1 and 872.7 eV are attributed to Ni^2+^ 2p_3/2_ and 2p_1/2_. After durability, the Ni 2p peaks (at 855.2 and 873.1 eV) shift toward the high binding energy while the peaks of S^2−^ 2p shift toward the low binding energy, suggesting electron transfer at the interface between Ni_9_S_8_ and NiO. In the O 1s XPS spectra (Fig. S6c), the peaks at 530.1 eV, 531.9 eV and 533.7 eV correspond to Ni–O, OH^−^ and absorbed H_2_O, respectively. After durability tests, the lattice oxygen (Ni–O) peak shifts to higher binding energy, indicating electrons flow from NiO to Ni_9_S_8_. Meanwhile, the Ni–O peak weakens while the OH^−^ peak strengthens, meaning surface hydroxylation of partial NiO. In an alkaline electrolyte, the surfaces of NiO and Ni(OH)_2_ are typically covered with hydroxyl species (–OH), which promotes the adsorption and dissociation of H_2_O molecules. The Ni site of nickel-based oxide and hydroxide favors the adsorption of the oxygen atom in H_2_O, thereby promoting the dissociation of the H–OH bond.^45,46^ Conversely, Ni_9_S_8_ with a higher electron density serves as favorable centers for H* adsorption/desorption. The synergistic catalysis among the active species enhances the HER performance of NiS-800@CNFs. Overall, the above proves that NiS-800@CNFs catalyst exhibits relatively good structural stability and electrochemical HER durability.

## 4 Conclusion

In summary, we achieved transformation from the sulfur-deficient Ni_9_S_8_ crystal to the sulfur-rich NiS crystal *via* adjusting the annealing temperature and thiourea content. The results confirm that the activity of the Ni_9_S_8_ phase is better than that of the NiS phase. With the increase of annealing temperature, the crystal structure of Ni_9_S_8_ becomes greatly obvious, the exposed active crystal surface is enhanced, and the degree of graphitization of the carbon fiber is enhanced. Specifically, the monolith NiS-800@CNFs electrode showed the best HER activity (*η*_10_ = 119 mV) and excellent durability under the high current density, contributed to the synergistic catalysis of NiO and Ni_9_S_8_ and the stable carbon-fiber frame. Through electrochemical impedance and phase diagrams, it is revealed that the two sulfur phase, NiS_*x*_ crystal follow the Volmer–Heyrovsky mechanism. The Volmer step is considered as an RDS process for the sulfur-deficient Ni_9_S_8_ phase, while the Heyrovsky step is used as the RDS process for the sulfur-rich NiS phase.

## Conflicts of interest

There are no conflicts to declare.

## Supplementary Material

NA-OLF-D5NA00797F-s001

## Data Availability

The data supporting this study and further experimental details are available within the supplementary information (SI). Supplementary information (SI) is available. See DOI: https://doi.org/10.1039/d5na00797f.
