# Supplementary material for: Controllable synthesis of nickel sulfides integrated with carbon fibers towards enhanced hydrogen evolution reaction kinetics
Source: Nanoscale Adv. 2026 Mar 5;8(8):2574–83. doi: 10.1039/d5na00797f (PMC12993534; doi:10.1039/d5na00797f)
Supplement: NA-008-D5NA00797F-s001 [file NA-008-D5NA00797F-s001.pdf]

Supplementary Information (SI) for Nanoscale Advances.
This journal is © The Royal Society of Chemistry 2026

Supplementary Information

Controllable synthesis of nickel sulfides integrated with carbon fibers towards enhanced hydrogen evolution reaction kinetic

Yuting Li [a, b], Juan Wang [*b], Qin Zhong [*b]

[a] College of Safety Engineering and Emergency Management, Nantong Institute of Technology, Nantong, Jiangsu, 226002, China

[b] School of Chemistry and Chemical Engineering, Nanjing University of Science and Technology, Nanjing, Jiangsu, 210094, China.

*Corresponding author

E-mail: wangjuan304@njust.edu.cn, zq304@njust.edu.cn

Characterization

The scanning electron microscopy (SEM) characterization was carried out via a FEI Quanta 250 FEG. X-ray power diffraction (XRD) patterns were obtained with a Cu Kα radiation on a Bruker D8 ADVANCE diffractometer (λ = 1.5604 Å). The Raman spectra was performed via Aramis confocal Raman microscope (λ= 532 nm). The elemental valence states of the samples were tested by X-ray photoelectron spectroscopy (XPS) with Al Kα as the X-ray source on a Thermo-VG Scientific Escalab 250Xi spectrometer. The nitrogen adsorption-desorption characterization was investigated with an ASAP2460 Micromeritics equipment at 77 K. High-resolution transmission electron microscopy (HRTEM) and elemental mapping analysis were collected via America FEI Talos F200i.

Electrochemical measurements

Electrochemical measurements were conducted on a CHI 760E electrochemical workstation at 25 ℃. The as-prepared catalyst directly was used as a working electrode, a

carbon rod as the counter electrode and a mercury/mercuric oxide electrode (Hg/HgO, filled with 1.0 M KOH) as the reference electrode. All potentials were referenced to the reversible hydrogen electrode (RHE) according to the equation: $E_{RHE}=E_{Hg/HgO}+0.059 \times pH + 0.098$ V. Linear sweep voltammetry (LSV) was carried out at a scan rate of 2 mV s^{-1}. The electrochemically active surface area (ECSA) of the catalyst was evaluated by measuring the double-layer capacitance (C_{dl}) with cyclic voltammetry. The stability performance was measured by chronopotentiometry method. EIS was measured within the voltage range of 0.1-1.0 V at frequency ranging from 10^5 Hz to 1 Hz to analyze reaction kinetic of HER.

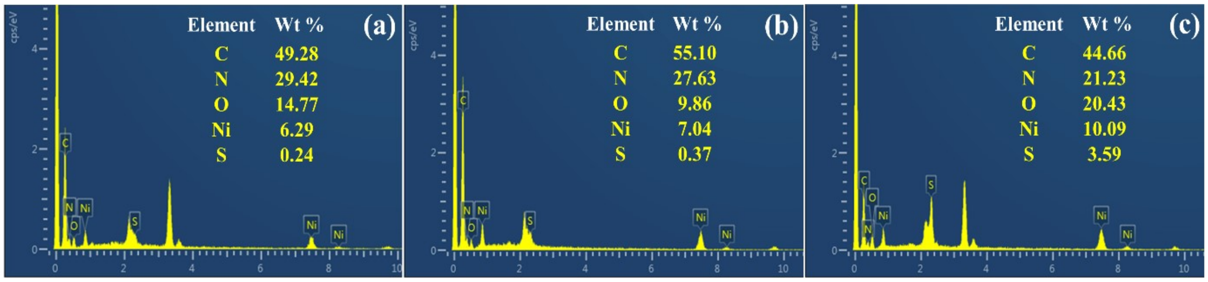

Fig. S1 EDX spectra of (a) NiS-0@CNFs, (b) NiS-1@CNFs and (c) NiS-2@CNFs.

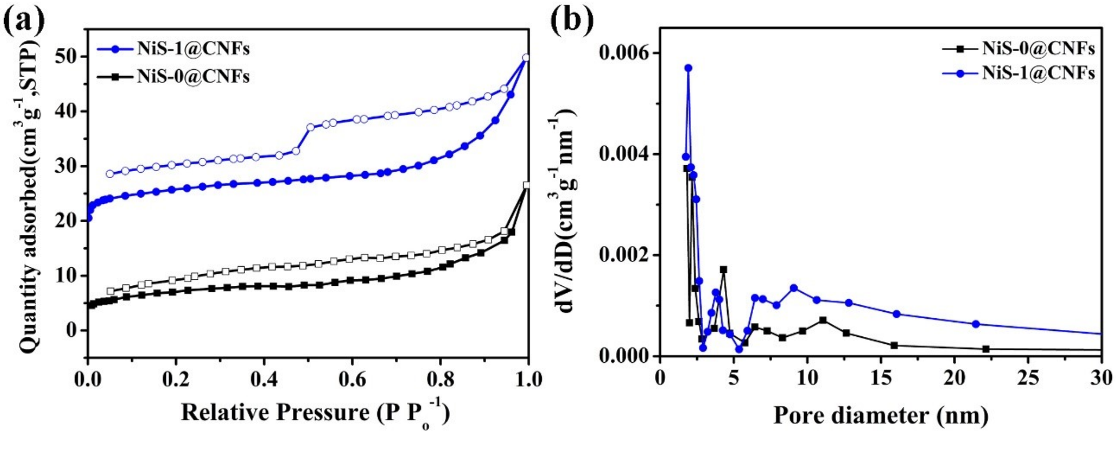

Fig. S2 N_2 adsorption/desorption isotherms (a) and pore size distribution (b) of NiS-y@CNFs

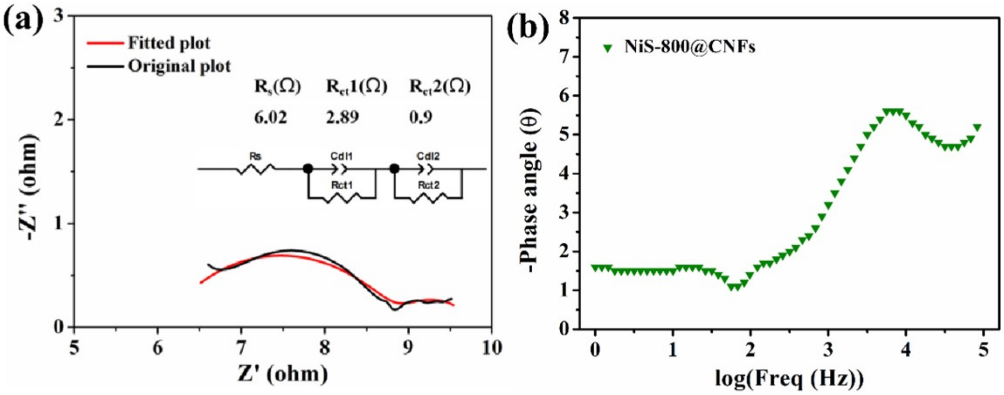

Fig. S3 Nyquist plot and Bode images of NiS-800@CNFs

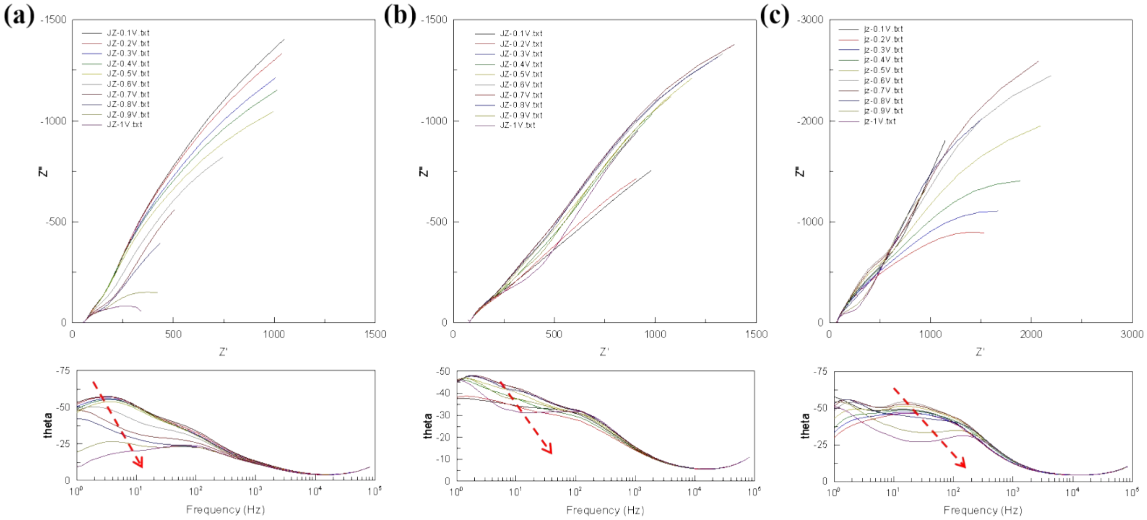

Fig. S4 Nyquist and Bode images at various overpotentials of NiS-0@CNFs(a), NiS-1@CNFs(b), and NiS-2@CNFs(c).

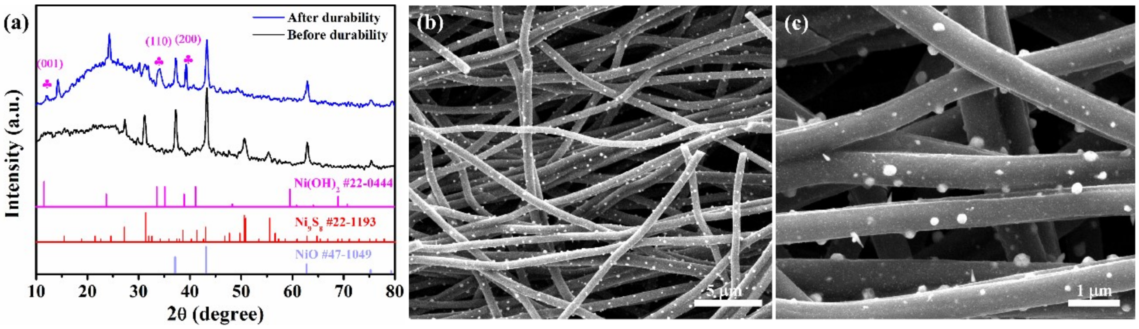

Fig. S5 (a) XRD pattern and (b, c) SEM images of NiS-800@CNFs after durability at high current density of 200 mA cm^{-2}.

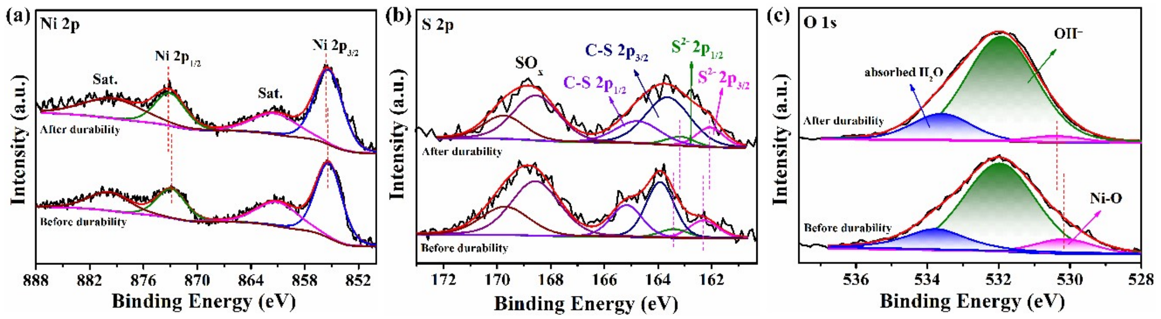

Fig. S6 XPS spectra of Ni 2p (a), S 2p (b) and O 1s (c) for NiS-800@CNFs after durability.

Table S1 Properties comparison of recent reported electrocatalysts for alkaline HER.

Catalyst	Overpotential at 10 mA cm^{-2} (mV)	Tafel slope (mV dec^{-1})	Testing substrate	Ref
NiS-800@CNFs	119	102.1	No-substrate	This work
NiS-ZnS/rGO-ST/NF	161	133	Silica template	1
CoS_x/Ni_3S_2@NF	204	133.32	Nickel foam	2
NiS-Ni_2P_2	147	68	GCE	3
Zn-NiS-3	208	113	Nickel foam	4
ZCS@rGO	135	47	Reduced graphene oxide	5
NiS/Mo_2CT_x	157	77	GCE	6
VS/Ni_xS_y/NF	125	113	Nickel foam	7
$NiFeVS_x$@NF	127	121	Nickel foam	8
MoS_2/CoS_2/CC	147	90.5	Carbon cloth	9
Mo-NiS_x/NF	155	88	Nickel foam	10

a. GCE: glassy carbon electrode

References:

1. Fetohi, A. E.; Khater, D. Z.; Amin, R. S.; El-Khatib, K. M., Nickel sulfide–transition metal sulfides bi-electrocatalyst supported on Nickel Foam for water splitting. *Journal of Physics and Chemistry of Solids* **2025,** *207*, 112906.

2. Shit, S.; Chhetri, S.; Jang, W.; Murmu, N. C.; Koo, H.; Samanta, P.; Kuila, T., Cobalt Sulfide/Nickel Sulfide Heterostructure Directly Grown on Nickel Foam: An Efficient and Durable Electrocatalyst for Overall Water Splitting Application. *ACS Applied Materials & Interfaces* **2018,** *10* (33), 27712-27722.
3. Das, J. K.; Sahu, N.; Mane, P.; Chakraborty, B.; Behera, J. N., A phase-engineered nickel sulfide and phosphide (NiS–Ni_2P) heterostructure for enhanced hydrogen evolution performance supported with DFT analysis. *Sustainable Energy & Fuels* **2023,** *7* (17), 4110-4119.
4. Prakash, C.; Sahoo, P.; Yadav, R.; Pandey, A.; Singh, V. K.; Dixit, A., Nanoengineered Zn-modified Nickel Sulfide (NiS) as a bifunctional electrocatalyst for overall water splitting. *International Journal of Hydrogen Energy* **2023,** *48* (58), 21969-21980.
5. Santhosh Kumar, R.; Ramakrishnan, S.; Prabhakaran, S.; Kim, A. R.; Kumar, D. R.; Kim, D. H.; Yoo, D. J., Structural, electronic, and electrocatalytic evaluation of spinel transition metal sulfide supported reduced graphene oxide. *Journal of Materials Chemistry A* **2022,** *10* (4), 1999-2011.
6. Wu, N.; Liu, J.; Zhao, W.; Du, J.; Zhong, W., Molybdenum carbide MXene embedded with nickel sulfide clusters as an efficient electrocatalyst for hydrogen evolution reaction. *International Journal of Hydrogen Energy* **2023,** *48* (46), 17526-17535.
7. Shang, X.; Yan, K.-L.; Rao, Y.; Dong, B.; Chi, J.-Q.; Liu, Y.-R.; Li, X.; Chai, Y.-M.; Liu, C.-G., In situ cathodic activation of V-incorporated Ni_xS_y nanowires for enhanced hydrogen evolution. *Nanoscale* **2017,** *9* (34), 12353-12363.
8. He, Y.; Shen, J.; Li, Q.; Zheng, X.; Wang, Z.; Cui, L.; Xu, J.; Liu, J., In-situ growth of VS_4 nanorods on Ni-Fe sulfides nanoplate array towards achieving a highly efficient and bifunctional electrocatalyst for total water splitting. *Chemical Engineering Journal* **2023,** *474*, 145461.
9. Fan, Y.; Yu, Y.; Sun, D.; Qu, Y.; Huang, L.; Wei, X.; Su, Q.; Du, G.; Xu, B.; Wang, K., Three-dimensional self-supporting micro-nanostructured MoS_2/CoS_2/CC heterojunction derived from ZIF-67 for high efficiency electrocatalytic hydrogen evolution in both acid and alkali electrolytes. *Journal of Alloys and Compounds* **2024,** *976*, 173354.
10. Zhang Y.; Chen M.; Guo P.; Du Y.;Song B.; Wang X.; Jiang Z., Xu P.; Magnetic field-enhanced water splitting enabled by bifunctional molybdenum-doped nickel sulfide on nickel foam. *Carbon Energy* **2023,** *5* (11), e351.
